# Association polymorphism of guanine nucleotide–binding protein β3 subunit (*GNB3*) C825T and insertion/deletion of the angiotensin-converting enzyme (*ACE*) gene with peripartum cardiomyopathy

**DOI:** 10.3389/fcvm.2023.1096514

**Published:** 2023-04-05

**Authors:** Ivana Purnama Dewi, Louisa Fadjri Kusuma Wardhani, Irma Maghfirah, Kristin Purnama Dewi, Agus Subagjo, Mochamad Yusuf Alsagaff, Johanes Nugroho

**Affiliations:** ^1^ Department of Cardiology and Vascular Medicine, Faculty of Medicine, Airlangga University—Dr. Soetomo General Hospital, Surabaya, Indonesia; ^2^ Faculty of Medicine, Duta Wacana Christian University, Yogyakarta, Indonesia; ^3^ Department of Pulmonology and Respiratory Medicine, Faculty of Medicine, Airlangga University—Dr. Soetomo General Hospital, Surabaya, Indonesia

**Keywords:** single nucleotide polymorphism, peripartum cardiomyopathy, PPCM, guanine nucleotide–binding protein subunit β3, angiotensin-converting enzyme

## Abstract

**Introduction:**

Peripartum cardiomyopathy (PPCM) is a potentially life-threatening pregnancy-related heart disease. Genetic roles such as gene polymorphisms may relate to the etiology of PPCM. This study analyzes the association between single nucleotide gene polymorphism (SNP) guanine nucleotide–binding protein beta-3 subunit (*GNB3*) C825T and insertion/deletion (I/D) of the angiotensin-converting enzyme (*ACE*) gene with the incidence of PPCM.

**Methods:**

An analytic observational study with a case–control design was conducted at the Integrated Cardiac Service Center of Dr. Soetomo General Hospital, Surabaya, Indonesia. PPCM patients of the case and control groups were enrolled. Baseline characteristic data were collected and blood samples were analyzed for SNP in the *GNB3* C825T gene and for I/D in the *ACE* gene by using the polymerase chain reaction, restriction fragment length polymorphism, and Sanger sequencing. We also assessed *ACE* levels among different *ACE* genotypes using a sandwich-ELISA test.

**Results:**

A total of 100 patients were included in this study, with 34 PPCM cases and 66 controls. There were significant differences in *GNB3* TT and TC genotypes in the case group compared with that in the control group (TT: 35.3% vs. 10.6%, *p* = 0.003; TC: 41.2% vs. 62.5%, *p* = 0.022). The TT genotype increased the risk of PPCM by 4.6-fold. There was also a significant difference in the *ACE* DD genotype in the case group compared with that in the control group (26.5% vs. 9.1%, *p* = 0.021). DD genotypes increased the risk of PPCM by 3.6-fold. *ACE* levels were significantly higher in the DD genotype group than in the ID and II genotype groups (4,356.88 ± 232.44 pg/mL vs. 3,980.91 ± 77.79 pg/mL vs. 3,679.94 ± 325.77 pg/mL, *p* < 0.001).

**Conclusion:**

The TT genotype of *GNB3* and the DD genotype of the *ACE* are likely to increase the risk of PPCM. Therefore, these polymorphisms may be predisposing risk factors for PPCM incidence. *ACE* levels were significantly higher in the DD genotype group, which certainly had clinical implications for the management of PPCM patients in the administration of *ACE* inhibitors as one of the therapy options.

## 1. Introduction

Maternal mortality ratio (MMR) is an indicator that describes national maternal health and welfare. Global MMR reached 214 per 100,000 live births in 2016 (1). In developing countries, MMR is 20 times higher than in developed countries (1). In 2012, Indonesia's MMR was 359 per 100,000 live births (2). An evaluation of the 2015 Millennium Development Goals revealed that 38 mothers in Indonesia died from diseases or complications related to pregnancy and childbirth every day (MMR: 305 per 100,000 live births). The causes of maternal death are mainly bleeding, infection, and cardiovascular disease, including hypertension during pregnancy and heart failure (3).

Peripartum cardiomyopathy (PPCM) is a potentially life-threatening pregnancy-related disease (4). PPCM is characterized by left ventricle (LV) dysfunction in the late peripartum period or in the first months of postpartum without a known history of heart disease (5). To date, there are many hypotheses about the etiology of PPCM, but none is considered as the primary explanation for all cases. PPCM is known to have a pathogenesis that involves many factors such as maternal autoimmune response, inflammation, oxidative stress, imbalance of cardiac proapoptotic factors and anti-angiogenic factors, micronutrient deficiencies, and genetic causes (6). Due to the complexity of the etiology, genetic factor, especially gene polymorphism, may play an essential role (7). Two major PPCM registries, Investigation of Pregnancy Associated Cardiomyopathy (IPAC) (8) and EURObservational Research Programme (EORP) (9), reported various incidence rates of PPCM among countries in different regions, which may be related to genetic predisposition in different races.

The guanine nucleotide–binding protein subunit β3 (*GNB3*) gene encodes the β3 subunit of G protein (Gβ3) located on chromosome 12p13 that consists of 11 exons and 10 introns. The single nucleotide polymorphism (SNP) of *GNB3* at exon 10, C825T, is associated with an increased prevalence and poor outcome of PPCM in individuals of African progeny (10). T allele polymorphisms in the *GNB3* gene are associated with increased intracellular signaling, increased risk of hypertension, low plasma renin, and cardiac remodeling (10). To date, there are no studies on *GNB3* C825T gene polymorphism, especially in Asian populations.

The role of the insertion/deletion (I/D) 287-bp sequence inside intron 16 of the angiotensin-converting enzyme (*ACE*) gene and *ACE* activity in the etiology, pathogenesis, prognosis, and clinical implications of the cardiovascular system has been extensively studied. The deletion polymorphism of the *ACE* allele is associated with increased levels of *ACE* (11). In addition, the *ACE* DD genotype is positively correlated with specific cardiomyopathy such as ischemic cardiomyopathy (ICM), hypertrophic cardiomyopathy (HCM), alcoholic cardiomyopathy, and idiopathic dilated cardiomyopathy (IDCM) (12, 13). IDCM with low ejection fraction (EF) has a phenotype similar to PPCM, suggesting that there may be an association between the I/D of the *ACE* gene and PPCM. This study aims to determine the association between the SNP of the *GNB3* C825T gene and the I/D of the *ACE* gene in women with PPCM.

## 2. Methods

### 2.1. Study design

An analytic observational study with a case–control study was conducted at the Integrated Cardiac Service Center, at Dr. Soetomo General Hospital and Institute of Tropical Diseases (ITD) Laboratory of Airlangga University, Surabaya, Indonesia from January 2021 to June 2022. The case group consisted of all women diagnosed with PPCM, while the control group comprised women without PPCM or a history of PPCM. The study was approved by the Dr. Soetomo General Hospital Surabaya Ethics Committee (0151/KEPK/II/2021). All procedures were approved by the relevant ethics committees and written informed consent was obtained from all study participants.

### 2.2. Patients and controls

All women who were 18–40 years’ old and who underwent examination and treatment at the Polyclinic Integrated Cardiac Service Center of Dr. Soetomo General Hospital Surabaya were included. PPCM was diagnosed according to the criteria of the European Society of Cardiology (ESC) Working Group on Peripartum Cardiology in 2010 (14). The criteria were: (1) Heart failure symptoms that appeared in the last 1 month of pregnancy to 5 months’ postpartum; (2) No history and other identifiable causes of heart failure; and (3) An left ventricular ejection fraction (LVEF) <45% based on echocardiography. All PPCM patients with previous history of heart failure, a history of coronavirus disease 2019 (COVID-19) infection complicated with any heart problems, and incomplete data were excluded. Controls were women with a history of pregnancy who had never been diagnosed with PPCM.

### 2.3. Detection of *GNB3* C825T gene polymorphisms and *ACE* gene I/D

Patients selected on the basis of inclusion and exclusion criteria and signed a letter of informed consent to participate in the study. A 5 mL sample of cubital venous blood was collected in an ethylenediaminetetraacetic acid (EDTA) tube and rested for about 30 min. The tube was then centrifuged at 300 rpm for 10 min to separate the plasma. DNA extraction was carried out using the QiaAMP DNA Blood Mini Kit (Qiagen, Hilden, Germany) and stored at −20°C. DNA content was quantified by spectrophotometric absorption (Nanodrop Spectrophotometer, BioLab, Scoresby, VIC, Australia). All DNA samples were blind-tested.

**Figure 1:**
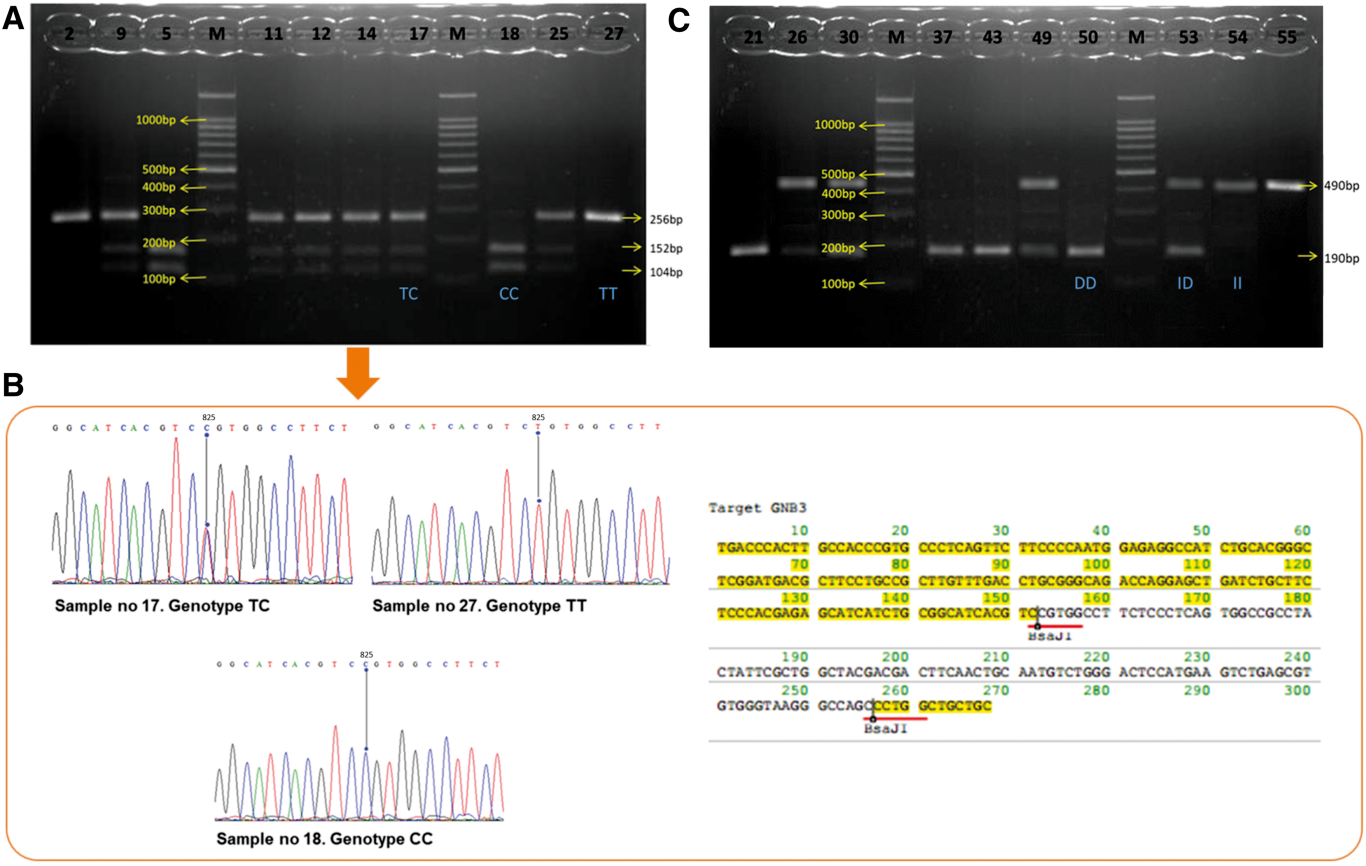
(**A**) Visualization of *GNB3* PCR products. The results from 10 samples show TT genotypes: sample nos. 2 and 27; CC genotypes: sample nos. 5 and 18; TC genotypes: sample nos. 9, 11, 12, 14, 17, and 25. (**B**) DNA sequencing chromatogram and amino acid sequence restriction of *GNB3*. The lines point to nucleotide 825. Chromatogram of *GNB3* shows TC genotypes at the upper left, TT genotypes at the upper right, and CC genotypes at the middle bottom. (**C**) Visualization of *ACE* gen PCR products. The results from 10 samples show DD genotypes: sample nos. 21, 37, 43, and 50; II genotypes: sample nos. 54 and 55; TC genotypes: sample nos. 26, 30, 49, and 53. *GNB3*, guanine nucleotide–binding protein beta-3 subunit; PCR, polymerase chain reaction; *ACE*, angiotensin-converting enzyme.

The *GNB3* C825T polymorphism was examined according to the procedure stipulated by Siffert et al. (15). We used 5′ TGACCCACTTGCCACCCGTGC 3′ as a sense primer and 5′ GCAGCAGCCAGGGCTGGC 3′ as an antisense primer. The polymerase chain reaction (PCR) was run using a Promega master mix reagent kit following the manufacturer's instructions (Promega, Madison, WI, United States). Amplifications were carried out in the T100TM Thermal Cycler (Bio-Rad, Hercules, CA, United States) as follows: 35 cycles with denaturation at 94°C for 1 min; annealing at 58°C for 45 s; extension at 72°C for 1 min; and a final extension at 72°C for 7 min. Amplification products were digested by using the restriction endonuclease enzyme, BseDI/BsaJI (MBI Fermentas, St. Leon-Rot, Germany), at 37°C in a water bath for 3 h and 80°C for 5 min. DNA fragments were obtained after the restriction enzyme was electrophoresed on a 2.5% agarose gel and stained with ethidium bromide and the BenchTop 1,000 bp DNA Ladder (Promega, Madison, WI, United States). The DNA fragments were imaged under ultraviolet (UV). The T allele was not digested by using the restriction endonuclease enzyme. It corresponded to the cDNA fragments of 256 bp (TT genotype), whereas the C allele corresponded to 152 bp and 104 bp (CC genotype). Thus, the CT genotype produced three bands, 256 bp, 152 bp, and 104 bp (Figure 1A). Three representative samples of each genotype (TT, TC, and CC) were confirmed with DNA sequencing using the Sanger method. DNA sequencing for the *GNB3* C825T polymorphism was performed by using the ABI Prism 24-capillary 3,500xL Genetic Analyzer to confirm the PCR result. The sequence analysis of the DNA is shown in Figure 1B. The results were compared with the reference strains of the sequences that were published in GenBank using the Clone Manager Professional version 9.0.

The I/D *ACE* polymorphism was examined as described by Rigat et al. (16). To amplify the *ACE*, a pair of primers 5′ CTGGAGACCACTCCCATCCTTTCT 3′ and an antisense primer 5′ GATGTGGCCATCTTCGTCAGA 3′ were used. The PCR amplification was processed as described for *GNB3*. The PCR product is a 490 bp fragment in the presence of the insertion (I) allele and a 190 bp fragment in the presence of the deletion (D) allele. Thus, each DNA sample revealed one of three possible patterns after electrophoresis: a 490 bp band (genotype II), a 190 bp band (genotype DD), or both 490 bp and 190 bp bands (genotype ID) (Figure 1C).

### 2.4. *ACE* ELISA test

Plasma from the PPCM group sample was separated after centrifugation and stored at −20°C until analysis. To determine the level of the *ACE* between different alleles and the genotype of *ACE* I/D, a sandwich-ELISA test was conducted using a Human Angiotensin-Converting Enzyme 1 ELISA (Elabscience, Hubei, China). The resulting optical density was read by using the BioRad ELISA Reader at 450 nm.

### 2.5. Statistical analysis

The data obtained were processed using SPSS (IBM Statistics 20.0) for Windows. The Hardy–Weinberg Equilibrium (HWE) was used to estimate the number of heterozygous and homozygous variant carriers in non-evolving populations on the basis of allele frequency. The *χ*^2^ test for the degree of freedom (dF) = 1 and a *p*-value = 0.05 were used to determine whether the observed genotypic distribution for *GNB3* and *ACE* agreed with the HWE. The genotypes and alleles of *GNB3* and *ACE* between the PPCM and the control groups were assessed using the *χ*^2^ test or Fisher's exact test according to the obtained data. Odds ratios (ORs) with a 95% confidence interval (95% CI) were determined to find the association of gene polymorphism intensity with disease. The normality of data was assessed using the Kolmogorov–Smirnov test. An independent Student’s t-test or a Mann–Whitney test was used for numerical data analysis of the two groups. For numerical data with >2 groups, analysis was performed using one-way ANOVA or the Kruskal–Wallis test as appropriate. Univariate and multivariate logistic regression analyses were done to determine whether gene polymorphism was the independent predictor of PPCM. Differences with *p*-values <0.05 were considered statistically significant.

## 3. Results

### 3.1. Characteristics of patients

A total of 100 patients were included in the study, of which 34 were PPCM patients and 66 controls, and the characteristics of the case and control group patients are presented in Table 1. The mean BMI was higher in the PPCM group than in the control group (29.02 vs. 26.96, *p* = 0.037). The number of patients who had preeclampsia or eclampsia was significantly higher in the PPCM group than in the control group (44.1% vs. 9.1%, *p* < 0.001). The PPCM group had a higher mean systolic blood pressure than the control group (143.26 mmHg vs. 131.09 mmHg, *p* = 0.007). A total of 91.2% of patients with PPCM were diagnosed antepartum (Table 1). We did not find any deviations from the HWE in our population study.

**Table 1:** Baseline characteristics of PPCM and controls.

Characteristics	PPCM (*n* = 34), *n* (%)	Control (*n* = 66), *n* (%)	*p*-value
Age, mean ± SD	29.09 ± 6.16	27.95 ± 5.72	0.362
BMI	29.02 ± 5.04	26.96 ± 4.09	0.037
Parity			0.155
Primiparous	15 (44.1)	39 (59.1)	
Multiparous	19 (55.9)	27 (40.9)	
Multifetal pregnancy	3 (8.8)	2 (3.0)	0.208
History of hypertension	6 (17.6)	4 (6.1)	0.085
Preeclampsia/eclampsia	15 (44.1)	6 (9.1)	<0.001
Systolic blood pressure, mean ± SD	142.03 ± 22.04	131.09 ± 18.69	0.007
Diastolic blood pressure, mean ± SD	84.97 ± 16.08	80.83 ± 11.65	0.144
Diagnosis time			NA
Antepartum	31 (91.2)	NA	
Postpartum	3 (8.8)	NA	

PPCM, peripartum cardiomyopathy; BMI, body mass index.

### 3.2. *GNB3* and *ACE* gene polymorphisms and the risk of PPCM

Of the total number of samples, the genotypes of *GNB3* were mostly TC (*n* = 57, 57%), followed by CC (*n* = 24, 24%) and TT (*n* = 19, 19%). There were significant differences in the frequency of TT and TC genotypes of the *GNB3* gene between the PPCM and the control groups (Table 2). Individuals with the TT genotype had a higher odds ratio of approximately 4.59 to have PPCM compared with those with the TC and CC genotypes (OR: 4.59; 95% CI: 1.60–13.17, *p* = 0.003) (Table 2). Although the frequency of T allele was higher in the PPCM group, the difference was not statistically significant compared with that in the control group (55.9% vs. 43.2%, *p* = 0.088).

**Table 2:** Comparison of genotype and allele frequencies of the *GNB3* and *ACE* genes in PPCM and control groups.

Gene	Comparison	PPCM *n* (%)	Control *n* (%)	OR	95% CI	*p*-value
*GNB3*	Genotype					
	TT vs. TC + CC	12 (35.3)	7 (10.6)	4.59	1.60–13.18	0.003
	TC vs. TT + CC	14 (41.2)	43 (65.2)	0.37	0.16–0.88	0.022
	CC vs. TT + TC	8 (23.5)	16 (24.2)	0.96	0.36–2.54	0.937
	Allele T vs. C					
	T	38 (55.9)	57 (43.2)	1.66	0.92–3.01	0.088
	C	30 (44.1)	75 (56.8)			
*ACE*	Genotype					
	DD vs. ID + II	9 (26.5)	6 (9.1)	3.60	1.16–11.18	0.021
	ID vs. DD + ID	7 (20.6)	20 (30.3)	0.59	0.22–1.59	0.300
	II vs. DD + ID	18 (52.9)	40 (60.6)	0.73	0.32–1.69	0.462
	Allele D vs. I					
	D	25 (36.8)	32 (24.2)	1.82	0.96–3.42	0.063
	I	43 (63.2)	100 (75.8)			

*GNB3*, guanine nucleotide–binding protein beta-3 subunit; *ACE*, angiotensin-converting enzyme; PPCM, peripartum cardiomyopathy.

Our data indicated that 15 subjects had the DD genotype, 27 had the ID genotype, and 58 the II genotype of the *ACE*. The DD genotype was significantly higher in the PPCM group than in the control group (26.5% vs. 9.1%), and the presence of the DD genotype was associated with a higher risk of PPCM compared with individuals with the ID and II genotypes (OR: 3.60; 95% CI: 1.15–11.18, *p* = 0.021) (Table 2). The frequency of D allele was higher in the PPCM group, but the difference was not statistically significant compared with that in the control group (36.8% vs. 24.2%, *p* = 0.063) (Table 2). Univariate and multivariate logistic linear regression analyses were done on various variables, as presented in Table 3. The analysis from Table 3 showed that *GNB3* TT and preeclampsia/eclampsia were independent predictors for PPCM.

**Table 3:** Predictor of the PPCM determinate by univariate and multivariate logistic regression analyses.

Factor	Univariate			Multivariate		
	OR	95% CI	*p*- value	OR	95% CI	*p*- value
BMI	1.81	0.73–4.48	0.199			
Parity	1.83	0.79–4.22	0.155			
Multifetal pregnancy	3.10	0.49–19.50	0.208			
History of hypertension	3.32	0.87–12.70	0.085			
Preeclampsia/ eclampsia	7.90	2.69–23.21	<0.001	7.55	2.49–22.88	<0.001
*GNB3* gene polymorphism	4.59	1.60–13.18	0.003	3.74	1.18–11.85	0.025
*ACE* gene polymorphism	3.60	1.16–11.18	0.021			

*GNB3*, guanine nucleotide–binding protein beta-3 subunit; BMI, body mass index; OR, odds ratio; CI, confidence interval; *ACE*, angiotensin-converting enzyme; PPCM, peripartum cardiomyopathy.

### 3.3. Subanalysis of the *GNB3* and *ACE* genotypes in the PPCM group

In PPCM patients, the frequency of the *GNB3* genotype was significantly different and was based on BMI and left ventricular internal diameter in diastole (LVIDd) (Table 4). The BMI was higher in the TT genotype of *GNB3* than in the TC and CC genotypes (31.73 vs. 27.54 kg/m^2^, *p* = 0.018). The mean of LVIDd was also higher in the TT genotype group than in the TC and CC groups (5.39 ± 0.80 vs. 4.86 cm ± 0.64 cm, *p* = 0.041). Hypertension and a history of preeclampsia/eclampsia were more frequent among those with the *ACE* DD genotype than among those with the ID and II genotypes; 44.4% vs. 8.0%, *p* = 0.031 and 77.8% vs. 32.0%, *p* = 0.025, respectively (Table 4).

**Table 4:** Comparison of *GNB3* and *ACE* gene genotypes in the PPCM group.

Variable	*GNB3*			*ACE*		
	TT *n* (%)	TC + CC *n* (%)	*p*-value	DD *n* (%)	ID + II *n* (%)	*p*-value
Hypertension						
Yes	3 (25.0)	3 (13.6)	0.641	4 (44.4)	2 (8.0)	0.031
No	9 (75.0)	19 (86.4)		5 (55.6)	23 (92.0)	
Preeclampsia/eclampsia						
Yes	4 (30.8)	11 (52.4)	0.296	7 (77.8)	8 (32.0)	0.025
No	9 (69.2)	10 (47.6)		2 (22.2)	17 (68.0)	
BMI (kg/m^2^), mean ± SD	31.73 ± 5.50	27.54 ± 4.20	0.018	29.88 ± 4.06	28.71 ± 5.40	0.559
Left ventricular ejection fraction (%), mean ± SD	36.92 ± 6.16	38.55 ± 5.50	0.437	36.78 ± 6.12	38.40 ± 5.61	0.506
Left ventricular internal diameter in diastole (cm), mean ± SD	5.39 ± 0.80	4.86 ± 0.64	0.041	5.12 ± 0.85	5.02 ± 0.71	0.726

*GNB3*, guanine nucleotide–binding protein beta-3 subunit; BMI, body mass index; *ACE*, angiotensin-converting enzyme; PPCM, peripartum cardiomyopathy.

### 3.4. Comparison of *ACE* levels based on *ACE* genotypes among PPCM patients

**Figure 2:**
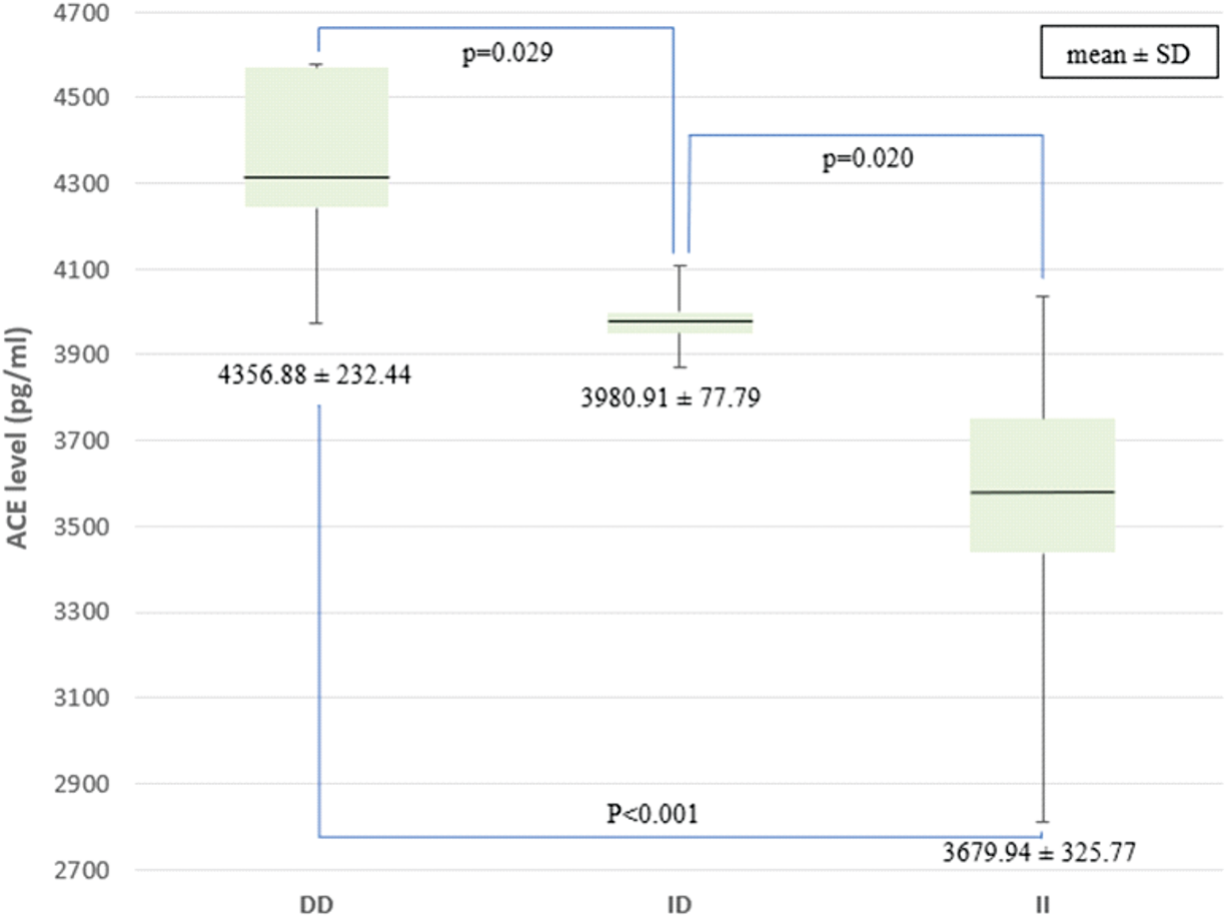
Comparison of *ACE* levels among the genotypes of the I/D *ACE* gene. *ACE*, angiotensin-converting enzyme; I/D, insertion/deletion.

The *ACE* levels were measured among 30 of 34 PPCM patients because four subjects received *ACE* inhibitors that may cause bias. Our data revealed that the *ACE* levels in DD, ID, and II were 4,356.88, 3,980.91, and 3,679.94 pg/mL, respectively. The *ACE* levels were significantly higher in the DD genotype group than in the ID and II genotype groups, *p* < 0.001. The *ACE* levels in individuals with the ID genotype were also higher than in individuals with the II genotype (*p* = 0.020) (Figure 2).

## 4. Discussion

Despite the growing recognition of genetic predispositions as a risk factor for the development of PPCM, little is known about the impact of genomic background on racial differences. Our study was the first one to determine whether there was an association of the SNP *GNB3* C825T gene and the incidence of PPCM in an Asian population. Although the frequency of the TT genotype was relatively rare, this genotype increased the risk of PPCM 4.6 times compared with the other CC and TC genotypes. Multivariate analysis also showed that *GNB3* TT appeared to be an independent predictor for PPCM. A study conducted in North America with 97 subjects (30% were Blacks, 65% were Caucasian, and 5% were others), which assessed the relationship between different *GNB3* genotypic backgrounds and their impact on improvement in LV remodeling in PPCM, found that the *GNB3* TT genotype was more common in Blacks (10). *GNB3* TT was also associated with a much higher incidence of PPCM and lower LVEF recovery (10).

Interestingly, our study found that the TC genotype (57%) was the most frequent genotype and may appear to afford protection from PPCM. However, there is limited evidence of the association between *GNB3* TC polymorphism and PPCM incidence. The exact mechanism by which the *GNB3* polymorphism contributes to the development of PPCM has not been fully understood, but it is thought to involve alterations in the G protein-coupled receptor (GPCR) signaling pathway (17). The *GNB3* protein plays a key role in the signaling pathways through GPCR that control the contraction and relaxation of heart muscle cells (17, 18).

In addition, our study found that the most common genotypes of *ACE* were II (58%), followed by ID (27%) and DD (15%). The DD genotype increased the risk of PPCM 3.6 times compared with the other genotypes (II and ID). This result was similar to that of a study by Yaqoob et al., which found that the DD genotype was possibly a predisposing and independent risk factor for the pathophysiology of PPCM in the Kashmiri Indian population (13). The frequency of the DD genotype and D allele was also significantly higher in the PPCM population (13). The DD genotype was associated with poorer left ventricle systolic function in terms of ejection fraction, dimension, and left ventricle end-systolic and end-diastolic volumes (13).

Preeclampsia and eclampsia appear to be independent predictors for PPCM, as revealed by multivariate analysis. The pathophysiology of preeclampsia and eclampsia related to PPCM is still poorly understood, but several hypotheses suggest that hemodynamic stress caused by preeclampsia can contribute to the worsening of this condition (19). The EORP study states that the global preeclampsia incidence rate as a comorbid PPCM is 25%. A further investigation reveals that the rate of incidence in the Asia Pacific population reaches 46% (9). A meta-analysis of 22 observational studies with a total of 979 samples also reveals that 22% of PPCM patients develop preeclampsia/eclampsia (20).

No previous studies have reported an association of the *GNB3* and *ACE* polymorphisms with hypertension and preeclampsia, specifically in the PPCM population. In our subanalysis, we found that the percentage of PPCM patients with hypertension (44.4% vs. 8%, *p* = 0.032) and a history of preeclampsia (77.8% vs. 32%, *p* = 0.025) was higher in the *ACE* DD genotype group than in the other genotype groups. A review study found that previous studies reported conflicting results, but the majority found that the DD genotype was associated with the incidence of hypertension and preeclampsia in pregnancy. A study of 121 pregnant women with a gestational age of 27–40 weeks reported a higher frequency of the DD genotype in the essential hypertension group than in the control group (21). A meta-analysis of 40 studies with a total of 3,977 cases and 7,065 controls concluded that the DD genotype increased the risk of preeclampsia compared with the DD and ID genotypes (52% vs. 17%), and D allele increased the risk of preeclampsia 1.29 times more than I allele (22).

Obesity is a risk factor for PPCM. Hemodynamic alterations, apoptosis, and inflammation are three potential causes of pathogenesis. Obesity causes excessive levels of circulating fat to alter blood volume, which increases stroke volume and stresses the LV wall, which, in turn, cause eccentric LV hypertrophy and, eventually, LV dysfunction (23). However, no previous studies have reported an association of the *GNB3* and *ACE* gene polymorphisms with obesity in the PPCM population. A study of Caucasian, Chinese, and Black populations reported that the TT genotype had a higher mean BMI than other genotypes (TC and CC) (24). In our study, similar results were obtained, where the mean BMI in the TT genotype was significantly higher than that in the *GNB3* TC and CC genotypes.

Although the mean LVEF in the *GNB3* TT and *ACE* DD genotype groups has been reported to be lower in previous studies (10, 13), our data suggested no significant difference. Our results are in line with those of other studies, which showed no statistically significant difference in LVEF in the *ACE* DD genotype, although the mean LVEF was lower in the *ACE* DD genotype (13). However, the IPAC study reported that PPCM patients with the *GNB3* TT genotype showed a lower LVEF at the initial stage of the study (10). After follow-up, LVEF was found to be significantly lower for *GNB3* TT subjects at 6 months (*p* = 0.007) and 12 months (*p* < 0.001).

The geometry and thickness of the heart wall, especially the LV, are associated with cardiovascular risk. Our study found that *GNB3* genotypes were associated with LVIDd, while the *ACE* was not. In contrast to our finding, a previous study by Poch et al., reported a lower mean of LVIDd in the *GNB3* CC genotype group than in the TT and CT genotype groups in the essential hypertension population (25). Another study by Mahmood et al., also reported that the *GNB3* TT genotype had a strong association with the incidence of LV hypertrophy (26). Similar to our study, a previous study found no difference in mean LVIDd among different genotypes of the *ACE* gene (13).

### 4.1. Comparison of *ACE* levels in the PPCM group

A study found that the I/D polymorphism of the *ACE* influenced the level of serum *ACE* in a healthy population (16). Observations of genetic polymorphisms may explain the interindividual variability in plasma *ACE*. In our study, the highest mean *ACE* levels were found in the DD group, followed by ID and II. This is in line with a study that found that the *ACE* levels increased twice as high in the DD genotype group as in the ID genotype group (16). Another study of pregnant women with hypertension in India reported significantly higher *ACE* levels in the DD genotype group than in the ID and DD genotype groups (27). The I/D of the *ACE* gene affects not only plasma *ACE* levels but also tissue *ACE* (28). Higher *ACE* levels would increase angiotensin II, which affects various systems, including the cardiovascular system (29). In the PPCM group, elevated *ACE* levels in the *ACE* DD genotype may be associated with the incidence of hypertension. An awareness on the part of clinicians about the existence of differences in *ACE* levels in each genotype will certainly have implications for the management of PPCM patients, one of which is the administration of *ACE* inhibitors as a therapy option in PPCM patients, especially those with the *ACE* DD genotype.

### 4.2. Limitations

The synergistic relationship between the *GNB3* and the *ACE* gene could not be assessed in this study. In the PPCM group, only two patients had polymorphisms of both genes, while the control group had none. In this study, we did not analyze the levels of improvement in LVEF function in PPCM patients, which could have provided a better understanding of the issue. The reason for this lack of analysis was that we could not ask these patients to visit the hospital for an echocardiography examination because of the restrictions imposed by the COVID-19 pandemic, which has thrown many facets of the healthcare system out of gear.

## 5. Conclusion

This is a study determining the association of *GNB3* C825T and *ACE* gene polymorphisms and the incidence of PPCM in an Asian population. The presence of the *GNB3* TT genotype increases the risk of PPCM 4.6 times, while the *ACE* DD genotype potentially increases the risk of PPCM by 3.6 times. A subanalysis on PPCM patients found that those with TT had a higher BMI and LVIDd and also that those with the DD genotype were more prone to have hypertension and preeclampsia/eclampsia. *ACE* levels were significantly higher in the DD genotype group than in the ID and II genotype groups. These findings highlight the importance of gene polymorphisms in PPCM and, therefore, might be used as predictors and management strategies in the future.

## Data Availability

The data analyzed in this study are subject to the following licenses/restrictions: The datasets used are available from the corresponding author on reasonable request. Requests to access these datasets should be directed to the corresponding author.
